# Effect of Babassu Natural Filler on PBAT/PHB Biodegradable Blends: An Investigation of Thermal, Mechanical, and Morphological Behavior

**DOI:** 10.3390/ma11050820

**Published:** 2018-05-16

**Authors:** Vinicius C. Beber, Silvio de Barros, Mariana D. Banea, Markus Brede, Laura H. de Carvalho, Ron Hoffmann, Anna Raffaela M. Costa, Elieber B. Bezerra, Ingridy D. S. Silva, Katharina Haag, Katharina Koschek, Renate M. R. Wellen

**Affiliations:** 1 Fraunhofer-Institut für Fertigungstechnik und Angewandte Materialforschung (IFAM), Wiener Straße 12, D-28359 Bremen, Germany; markus.brede@ifam.fraunhofer.de (M.B.); ron.hoffmann@ifam.fraunhofer.de (R.H.); katharina.haag@ifam.fraunhofer.de (K.H.); katharina.koschek@ifam.fraunhofer.de (K.K.); wellen.renate@gmail.com (R.M.R.W.); 2 Faculty of Production Engineering, University of Bremen, Badgasteiner Straße 12, D-28359 Bremen, Germany; 3 Centro Federal de Educação Tecnológica Celso Suckow da Fonseca, Av. Maracanã 229, Rio de Janeiro, RJ 20271-110, Brazil; silvio.debarros@gmail.com (S.d.B.); mdbanea@gmail.com (M.D.B.); 4 Department of Materials Engineering, Federal University of Campina Grande, R. Aprígio Veloso 882, Campina Grande, PB 58249-140, Brazil; heckerdecarvalho@yahoo.com.br (L.H.d.C.); raffaela_matos@yahoo.com.br (A.R.M.C.); elieberbarros@hotmail.com (E.B.B.); 5 Department of Materials Engineering, Federal University of Paraíba, Cidade Universitária s/n, João Pessoa, PB 58051-085, Brazil; ingrydy.dayane@gmail.com

**Keywords:** PBAT/PHB/Babassu, cost-effective compounds, biodegradability, green composites, biomaterial, organic filler, mechanical characterization, differential scanning calorimetry, scanning electron microscopy

## Abstract

Blending of biodegradable polymers in combination with low-price organic fillers has proven to be a suitable approach to produce cost-effective composites in order to address pollution issues and develop products with superior mechanical properties. In the present research work PBAT/PHB/Babassu composites with 25, 50, and 75% of each polymer and 20% of Babassu were produced by melting extrusion. Their thermal, mechanical, and morphological behavior was investigated by differential scanning calorimetry (DSC), tensile testing, and scanning electron microscopy (SEM). Blending PBAT with PHB inhibited the crystallization of both polymers whereas adding Babassu did not significantly change their melting behaviour. Incorporation of Babassu reduced the tensile strength of its respective blends between 4.8 and 32.3%, and elongation at break between 26.0 and 66.3%. PBAT as highly ductile and low crystalline polymer may be seen as a crystallization tool control for PHB as well as a plasticizer to PBAT/PHB blends and PBAT/PHB/Babassu composites. As PBAT content increases: (i) elongation at break increases and (ii) surface fracture becomes more refined indicating the presence of more energy dissipation mechanisms. As PBAT/PHB/Babassu composites are biodegradable, environmental friendly, and cost effective, products based on these compounds have a great potential since their mechanical properties such as ductility, stiffness, and tensile strength are still suitable for several applications even at lower temperatures (−40 °C).

## 1. Introduction

Blending of biodegradable polymers as well as using low-price organic fillers aiming to produce cost-effective composites, besides being an alternative to solve pollution issues, might be a method of developing products with superior mechanical properties. The right combination of polymers and fillers can provide compounds with optimized properties for specific applications.

The development of such products has attracted considerable attention as environment friendly materials [1]. Bio-polyesters are among commercially available biodegradable polymers as they display properties similar to non-biodegradable polymers. For instance, polycaprolactone (PCL), polyhydroxybutyrate (PHB), poly(3-hydroxybutyrate-*co*-3-hydroxyvalerate) (PHBV), poly(lactic acid) (PLA), poly(butylene succinate) (PBS), poly(butylene succinate-co-adipate) (PBSA), and poly(butylene adipate-*co*-terephatalate) (PBAT) are currently being used in the plastic industry in several applications, such as packaging, electro-electronic, biomedical, food containers, and agricultural products [2,3,4].

In the present work, PHB/PBAT blends were melt mixed in a laboratory internal mixer, and afterward, composites with Babassu filler based on these blends were melting extruded. PHB used in this work is a biodegradable and biocompatible semi-crystalline polymer obtained from bacterial fermentation of sugarcane. Although PHB has similar mechanical properties to those of polyolefins such as polypropylene (PP) [5], its widespread application is limited due to high cost and certain inferior material properties including brittleness and low impact resistance, as well as poor thermal stability during processing [6]. 

In order to overcome these disadvantages, PBAT, a synthetic and biodegradable random copolymer of butylene adipate and terephthalate that has high thermal stability, elongation at break (as high as 700%), wear, and fracture resistance [3], was blended with PHB. However, PBAT displays low stiffness and strength, and therefore, PHB may improve these properties. 

Moreover, the natural and cost-effective Babassu filler (*Orbignya phalerata Mart. (Arecaceae*)) was added to the PHB/PBAT blends. Babassu is a palm tree naturally found in the North, Northeast and Midwest regions of Brazil. Its fruits are composed by a protective shell rich in lignin and cellulosic material. Usually, the shell is discarded and burnt, i.e., it is a material with no added value. Therefore, its use in polymer composites, beyond scientific and technological improvements can yield economic benefits. One of the aims of the present work is to propose potential applications with increased added value to this Brazilian filler [7].

Thermal properties of PBAT/PHB blends and Babassu composites were investigated by Differential Scanning Calorimetry (DSC). Their mechanical properties were determined at both room temperature (23 °C) and at low temperature (−40 °C). In addition, phase behavior analysis, i.e., dispersion/coalescence as well as the study of fracture mechanisms was performed by scanning electron microscopy (SEM).

## 2. Materials and Methods 

### 2.1. Materials

The polymers used were: (i) poly (butylene adipate-co-terephthalate) (PBAT), commercially available as BASF Ecoflex^®^ F Blend C1200 (Ludwigshafen, Germany), and (ii) poly (3-hydroxybutyrate) (PHB) commercially available as Biocycle^®^ 1000 supplied by PHB Industrial SA (Pedra Serrana, SP, Brazil). Both polymers were used without any further treatment.

The vegetable filler used was Babassu mesocarp, obtained from the fruit of Babassu palm tree (*Orbignya* sp.) supplied by Florestas Brasileiras S.A. (Itapecuru-Mirim, MA, Brazil) as a micronized powder. Babassu filler was used as received, and it is composed of cellulose (45%), hemicellulose (34%), and lignin (18%) with 3% of mineral ashes [1]. Babassu filler and PHB were dried in a vacuum oven for 4 h at 60 °C prior to processing.

### 2.2. Specimen Processing

Three PBAT/PHB blends containing 25%, 50% and 75% of each component were prepared. Composites containing 20% Babassu filler for each of the three blends as matrices were also processed. The composition of polymer blends and composites including sample codes are presented in Table 1. PBAT/PHB blends were compounded in a Haake Rheomix 3000 laboratory (Thermofisher, Waltham, MA, USA) internal mixer fitted with high intensity rotors (Rheochord 600, Thermofisher, Waltham, MA, USA). During processing, the chamber temperature was kept at 180 °C, the nominal rotor speed was set to 60 rpm and the material was processed for 10 min with a 70% fill factor at ambient temperature which corresponds to a total mass of 275 g. PBAT/PHB/Babassu composites were melting extruded in a twin-screw extruder NZ PHYPOLYMER SGS (diameter 22 mm and L/D ratio of 38, NZ Philpolymer, Sao Roque, SP, Brazil). 

Extruder barrel temperature was kept at 170 °C, and the nozzle temperature was 230 °C. After mixing and extrusion, the material was granulated and oven dried for 24 h at 40 °C. Injected specimens for mechanical tests (ASTM D638 standard) were produced in Arburg Allrounder 270A machine (Arburg, Lobburg, Germany) operating with a 150–180 °C temperature profile.

### 2.3. Tensile Experiments

Tensile tests were performed at room temperature (~23 °C) and at −40 °C. Prior to testing the samples were stored in a cabinet and into the tensile machine cold chamber, respectively, to ensure the thermal equilibrium. Tests were conducted under a displacement rate of 5 mm/min at both temperatures 

For experiments conducted at room temperature, the equipment employed was an INSTRON 5966 universal testing machine (INSTROM, Norwood, MA, USA)with electromechanical control and a load cell of 10 kN A clip-on extensometer (INSTROM 2630-107, INSTROM, Norwood, MA, USA) with gauge length (L_0_) of 25 mm was clipped onto the sample to measure local deformation. Experiments at −40 °C were carried out in a Zwick-Roell DO614728 (Zwick-Roell, Ulm, Germany) machine with a load cell of 10 kN with deformation values (i.e., strain) measured taking into account the cross-head displacement. 

### 2.4. Scanning Electron Microscopy (SEM) Analysis

Scanning Electron Microscopy analysis was performed to evaluate the morphological behaviour of blends and composites, i.e., interaction between PBAT, PHB and Babassu filler (phase dispersion/coalescence). 

Five samples were selected for this analysis. Two types of fractured surfaces were investigated: (i) three samples were analyzed after cryo-fracture (liquid nitrogen bath followed by brittle fracture), to assess the fracture behaviour with none/little plastic deformation, and (ii) two sample surfaces were analyzed directly after tensile testing (i.e., with elastic and plastic deformation). The description of sample composition, testing temperature, and fracture type are shown in Table 2.

SEM images were taken using a microscope SSX 550 Superscan (Shimadzu Corporation, Kyoto, Japan). On gold-sputtered samples, sputtering was carried out on a Shimadzu IC50 apparatus operating for 3 min with 10 mA of current, which provided an expected coating thickness of 50–100 nm. 

### 2.5. Differential Scanning Calorimetry (DSC) Measurements 

Thermal characteristics of PBAT/PHB blends and PBAT/PHB/Babassu composites were measured in a DSC Q20 differential scanning calorimeter (TA Instruments, New Castle, DE, USA) under nitrogen flow of 50 mL/min. Samples of approximately 4 mg were heated at 10 K/min from −90 °C to 250 °C. The melting parameters, i.e., temperatures, rates, enthalpies, and degree of crystallinity, are presented in Table 3. 

The degree of crystallinity ∆X_m_ was estimated taking into account the heat of melting of PBAT and PHB with 100% crystallinity according to Equation (1).

(1) $${\Delta X}_{m}=\frac{\Delta H}{{\Delta H}_{m}^{0}}$$

where ∆H is the melting enthalpy of PBAT or PHB and ${\Delta H}_{m}^{0}$ is the equilibrium melting enthalpy of these polymers, 114 J/g [8] and 146 J/g [9], respectively.

## 3. Results and Discussion

### 3.1. DSC Measurements of PBAT/PHB Blends

**Figure 1:**
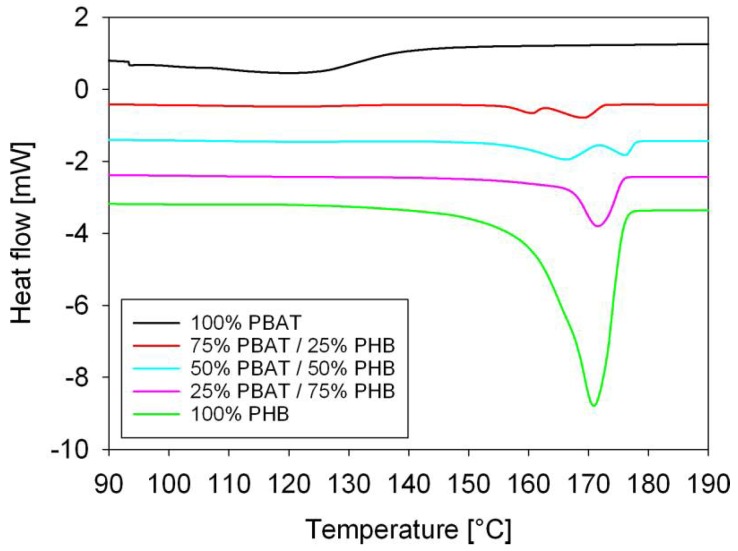
Differential scanning calorimetry (DSC) melting scans of PBAT, PHB, and PBAT/PHB blend. Heating rate of 10 K/min.

Thermal characterization of the neat polymers and of PBAT/PHB blends was performed by DSC. Figure 1 illustrates scans of the neat polymers and blends investigated in this work and their melting parameters are presented in Table 3 (first—lower temperature peak—PBAT) and Table 4 (second—higher temperature peak—PHB). DSC of neat PBAT and PHB displayed endotherms associated to melting of their crystalline phase. PBAT is less crystalline than PHB, so its melt peak is characterized by a flatter and wider band, which ranged between 89.35 and 142.61 °C and enthalpy of 18.64 J/g corresponding to a degree of crystallinity of 16.35%. PHB showed an endothermic peak between 146.68 and 177.90 °C and a degree of crystallinity of 60.89%. The degree of crystallinity measured for PBAT and PHB are within the range found in the literature [2,10]. Complex peaks and shoulders were observed during PHB melting, which is typical for PHB-based compounds as reported by Wellen et al. [11].

The melting peak of PBAT in the blends was displaced by 12.57 °C higher than in the neat PBAT. It is likely that heat transfer mechanisms were hampered in the blends. Prior to heating, the macromolecules and crystallites of PHB and PBAT are in a mixed-interlaced-solid state. At the beginning of PBAT melting, PHB chains and crystals are still solid. Possibly, this hinders the heat transfer and free movement of macromolecular chains contributing to the increase in energy required to melt PBAT [12]. Therefore, in order to melt PBAT/PHB blends more heat should be provided to the blend systems. Hence, their solid state is kept at higher temperatures which could be interesting for their mechanical performance, particularly when projecting products to support high temperatures. A detailed description of the mechanisms of heat transfer in blends was reported by Groeninckx et al. [12]. 

Blending PBAT with PHB inhibited the crystallization of both polymers as seen by the lowering in their degree of crystallinity (∆X_m_) presented in Table 3, where ∆X_m_ of PBAT decreased from 16.35% to 1.54%, while ∆X_m_ of PHB decreased from 60.89% to 19.79%. Previous DSC studies [13,14] of these blends indicated that melt crystallization of PHB ranges between 39.0 and 67.3 °C, whereas the PBAT ranges between 81.4 and 53.2 °C. 

Therefore, at the onset of the melt crystallization, PHB is in contact with a second heterogeneous liquid/solid phase—originated from the partial melt crystallized PBAT—whereas the PBAT starts melt crystallizing in contact with molten PHB. The chains of both polymers at the front or around the growing crystallites are slowing down and even preventing crystal growth. Thermal movements of chains are hindered and more energy must be supplied to the system so that crystallization takes place. Together these phenomena would be leading to less perfect crystals as well as to a lower degree of crystallinity. In the blends, spatial constraints hinder crystal growth, which prevents high degrees of crystallinity and in turn modifies the processes of melting and crystallization [15]. The whole system is much more complex than that of neat polymers. 

DSC scans of 75%PBAT/25%PHB and 50%PBAT/50%PHB displayed peaks associated to the melting of the neat polymers. In the lower temperature region, it is due to PBAT which has a single peak, and at higher temperatures, PHB melts as complex peaks. For the 25%PBAT/75%PHB blend no endotherm is observed in the PBAT melting range. This could be attributed to PHB hindering PBAT crystallization, thus supressing PBAT melting peak. On the other hand, the melting peak associated to PHB ranged between 141.34 and 176.12 °C, with enthalpy of 33.73 J/g and ∆X_m_ 23.10%. 

After processing, the melt crystallization of PHB during cooling takes place in an amorphous/crystalline region. It is believed that the presence of PBAT, even in low concentration, disturbs PHB crystallization lowering its degree of crystallinity. Blending leads to higher amorphous content which, in turn, will promote changes in the mechanical behavior as later discussed. 

These findings suggest that addition of PBAT to PHB can be adopted as way to control PHB crystallinity and vice versa. Similar results were obtained by Carofiglio et al. [16] in PHB/Olive mill wastewater (OMW) compounds as well as by Przybysz et al. [17] in PCL/PHB blends upon addition of dicumyl peroxide (DCP) and di-(2-*tert*-butylperoxyisopropyl)-benzene (BIB). In these works, it was found that the tensile strength and elongation at break were improved upon blending making PHB compounds suitable for agriculture and packing markets.

Regarding the present work, it is suggested the neat polymers behave as an anti-nucleant during their mutual crystallization as PBAT/PHB are phase separated blends. This means that the high crystallinity of PHB can be decreased and controlled upon PBAT addition. As recently reported, Wojtczak et al. [18] succeeded to control the crystallizability of PHB by blending with aliphatic-aromatic copolyester to such an extent that only a small fraction of the material crystallizes during cooling. They produced phase separated blends, and the crystallization of PHB in blends was strongly retarded as compared to pure PHB. According to the authors, this was caused by a dramatic decrease in number of primary nuclei initially present in PHB that migrated to copolyester component during compounding. 

Regarding the PHB melting endotherms, it is believed that the complex peaks appearing during PHB melting originated from [19]: (i) melting, recrystallization and re-melting during heating, (ii) the presence of more than one crystal modification (polymorphism), (iii) different morphologies (lamellar thickness, distribution, perfection or stability), (iv) physical aging or/and relaxation of the rigid amorphous fraction, or (v) different molecular weight species.

Since PHB starts melt crystallizing later, it is still in the melt state when PBAT is crystallizing, which might act as a diluent for PBAT. A portion of PHB nuclei will be in contact with PBAT crystallites and start growing along and between them, so that the amorphous solid phase and liquid (molten) phase will be also present. It is suggested that PHB crystallizes in regions with distinct spatial constraints, thus leading to crystals with different stabilities. These phenomena are more evident for blends with higher PBAT content (50% and 75%). This process would be the driving mechanism for the complex melting behavior observed in Figure 1.

Gunaratne and Shanks [20] attributed the multiple melting behavior of PHB to different crystal morphologies. In their work the hydroxyvalerate (HV) content would yield different crystallizable PHB, i.e., compounds with several degrees of crystallinity leading to complex melting. PHB compounds also undergo melting–recrystallization–remelting processes during heating, and by optical microscopy, it was revealed that sizes of spherulites were significantly increased as HV content increased. In another contribution Modi et al. [21] showed that HV broadens the PHB processing window allowing lower processing temperatures and improving melt stability. Multiple melting behaviors associated to different crystallinities were observed, which were responsible for changes in the mechanical performance.

**Figure 2:**
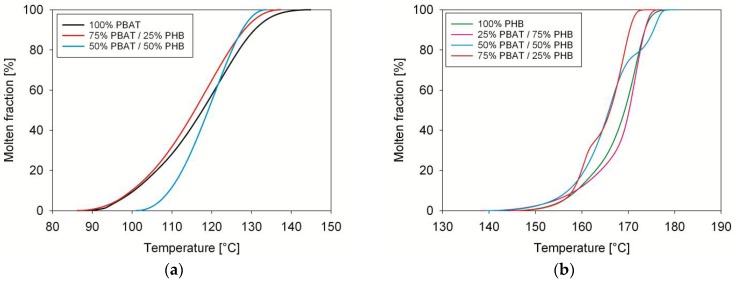
Molten fraction of PBAT/PHB blends: (**a**) lower temperature peak (PBAT); (**b**) right side higher temperature peak.

The molten fraction versus temperature of PBAT/PHB blends is displayed in Figure 2. Molten fraction attributed to PBAT is shown on the left side whereas the PHB is on the right side. The plots have a sigmoidal shape characteristic of phase transition without discontinuities. Apparently, blending did not change the melting characteristics of PBAT or PHB. Deviation from sigmoidal trend is observed for 50%PBAT/50%PHB and 75%PBAT/25%PHB, which might be associated to the complex melting behaviour of PHB. Similar trends were previously observed by Gunaratne and Shanks [22], and by Righetti and Di Lorenzo [23]. The thermodynamics and kinetics parameters of PBAT/PHB blends were computed using a custom software and are displayed in Table 3 and Table 4.

**Figure 3:**
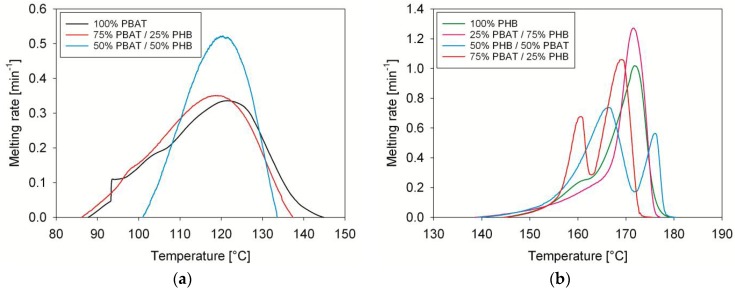
Melting rates of PBAT, PHB, and PBAT/PHB blends: (**a**) first peak; (**b**) second peak.

Figure 3 shows the melting rates of PHB, PBAT, and PBAT/PHB blends. The melting rate of PBAT increased with PHB addition indicating a less time-consuming process. However, it should be noted that the degree of crystallinity of PBAT in this blend is very low, around 1.54%. Except for the 50%PBAT/50%PHB blend that exhibited a lower melting rate, no significant changes were observed for PHB melting rates. Yeo et al. [24] discussed extensively the modification and melting behavior of PHB. On the other hand, literature database is scarce with regards to works dealing with the melting kinetics of PBAT/PHB compounds, which is the one of the insights brought by the present contribution. 

Melting half times (τ_½_,) were calculated from the sigmoidal curves in Figure 2, and its values are shown in Table 3. In general, τ_½_ decreased with decreasing of PBAT contents, which means that despite the increase in the melting temperature of PBAT, its melting is faster upon blending. Meanwhile, an increase in τ_½_ was observed for blends with increasing PHB content in the blends, indicating a slower melting process. 

### 3.2. DSC Measurements of PBAT/PHB/Babassu Composites

**Figure 4:**
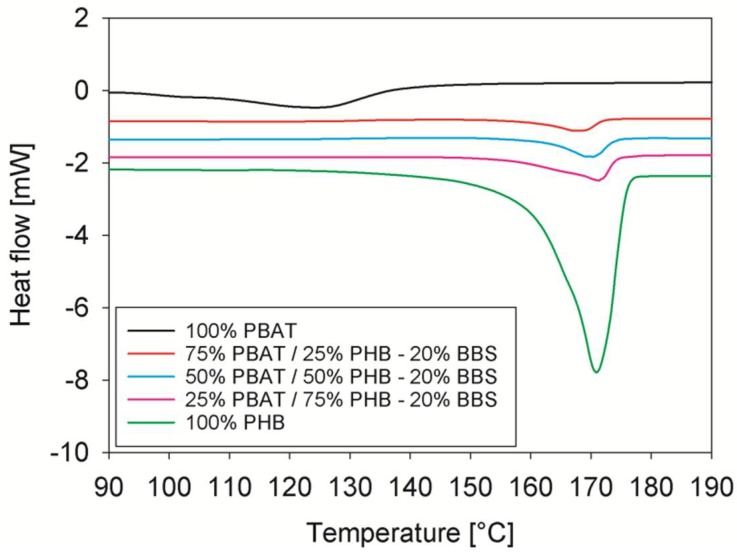
DSC scans of PBAT, PHB, and PBAT/PHB/Babassu composites. Heating rate of 10 K/min. BBS: Babassu.

DSC melting scans of PBAT/PHB/Babassu composites are displayed in Figure 4 and their melting parameters are shown in Table 5. Adding Babassu to PBAT/PHB blends did not significantly change the melting behaviour of PHB, which is observed as complex as described in the previous section. However, the melting peak attributed to PBAT melting was not observed for the composites. 

The effects of Babassu incorporation on the thermal and kinetics parameters of PBAT/PHB blends indicate that (i) melting temperatures of composites did not change considerably whereas (ii) the degree of crystallinity was reduced in the composites leading to the absence of PBAT endotherms in the DSC scans. Lim et al. [25] investigated polylactide (PLA)/poly(3-hydroxybutyrate-*co*-3-hydroxyhexanoate) (PHB-HHx) systems and showed that the crystallization of PLA was gradually suppressed by increasing PHB-HHx content. Results confirmed that the ductility of PHB-HHX and its inhibiting effect on PLA crystallization improved the stiffness of the systems compared to those of neat PLA. 

**Figure 5:**
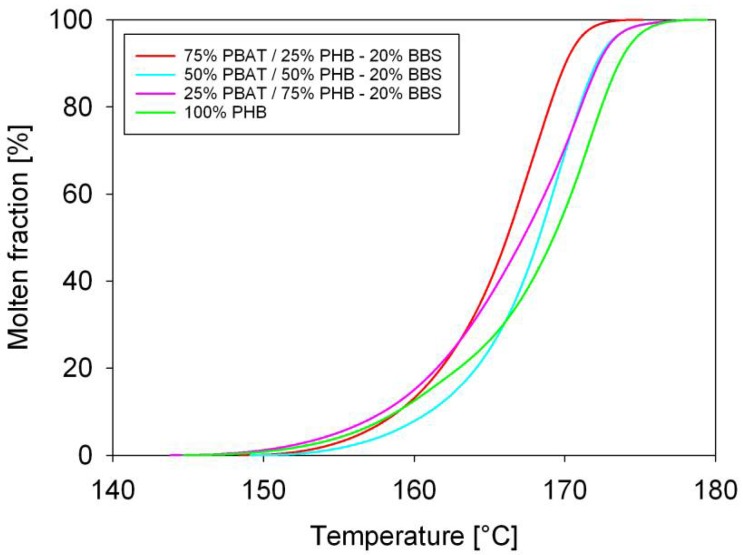
DSC scans of PBAT, PHB, and PBAT/PHB/Babassu composites. Heating rate of 10 K/min. BBS: Babassu.

In the present contribution, the suppression of PBAT and PHB crystallinity is likely responsible for the improvement of the mechanical response, particularly at low temperatures. Babassu increased the melting rate of the composites when compared to the blends (see Table 4). A similar trend was observed during the crystallization of PLA/Babassu compounds in which Babassu improved the crystallization rate as it acted as a nucleating agent decreasing the activation energy for the cold crystallization of PLA [14]. DSC peaks displayed in Figure 4 were integrated using a custom software and the plots are shown in Figure 5.

Figure 5 shows the molten fraction of PBAT/PHB/Babassu composites. It can be seen that the samples displayed a sigmoidal shape due to a phase transition without discontinuities. An overlapping of the curves is observed. These results suggest that the addition of 20% Babassu does not significantly modify the melting behavior of PBAT/PHB blends. 

Therefore, from the perspective of thermal behavior it is possible to produce cost effective PBAT/PHB/Babassu composites with up to 20% of Babassu. As Babassu is an inexpensive filler, the composites presented in this work are not only biodegradable but also cost-effective [26,27]. However, it is important to highlight that issues related to the processing of samples with different percentages of Babassu might affect this behavior. 

**Figure 6:**
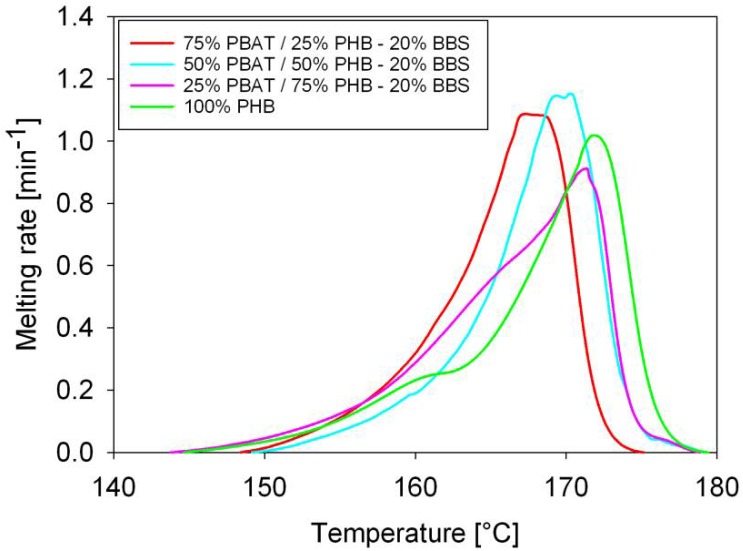
Melting rates of PHB and PBAT/PHB/Babassu composites. BBS: Babassu.

The melting rates of PBAT/PHB/Babassu composites are shown in Figure 6 and clearly show that all compositions presented very similar behaviour. However, the 50%PBAT/50%PHB/20%Babassu composite presented *c_max_* higher than the blend with similar composition (values were measured considering PHB melting peak).

Complex peaks and shoulders are observed in the region of lower temperature, i.e., starting of melting and may be originated due to the presence of heterogeneous crystals perfection as previously mentioned. 

### 3.3. Mechanical Properties of PBAT/PHB Blends

**Figure 7:**
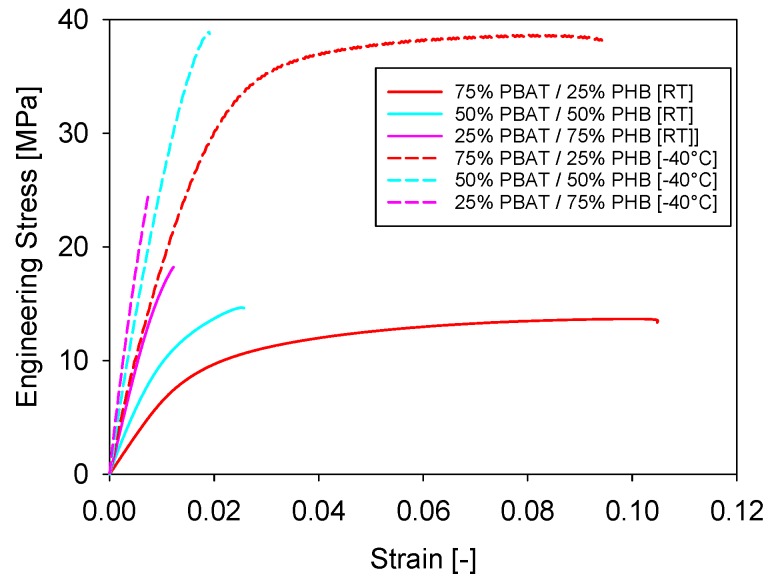
Representative stress-strain curves of PBAT/PHB blends at RT and −40 °C.

Representative stress-strain curves of PBAT/PHB blends performed at room temperature and at −40 °C are shown in Figure 7, where it is shown that as PBAT content increases the elongation at break of blends is increased. In this regard, a similar behaviour was observed on reactively compatibilized PHB/EVA/starch blends [28]. 

As expected, the tensile strength and Young’s Modulus increases with PHB content in the blends (Figure 7). These results are supported by DSC analysis, which shows that the degree of crystallinity of PHB is much higher than PBAT. In the crystalline region, where molecular chains are closely packed, the secondary links are stronger than in the amorphous region. Therefore, higher energy needs to be provided to the system for deformation to occur increasing their tensile strength.

**Figure 8:**
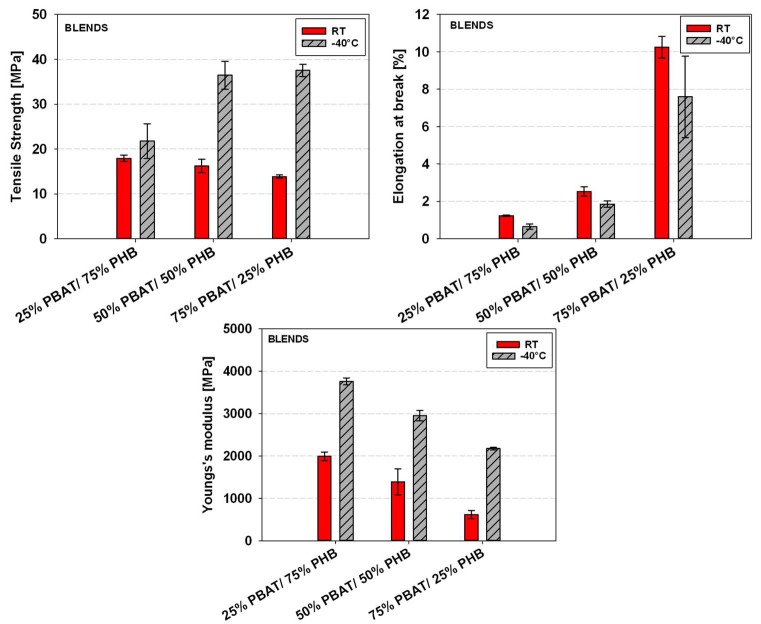
Effect of temperature on the mechanical properties of PBAT/PHB blends.

Figure 8 shows a comparison between the mechanical properties of PBAT/PHB blends tested at room temperature and at −40 °C. Results reported are an average of five tests.

Young’s Modulus and tensile strength values were shown to be higher and elongation at break to be lower for samples tested at −40 °C when compared with the data taken at room temperature (Figure 8). At −40 °C, a temperature below the T_g_ of both PBAT and PHB, the free movement and deformation mechanisms of both macromolecules are more restricted. Nevertheless, the maximum deformation at break apparently did not significantly change at lower temperatures. 

These are desirable properties in determining potential applications of blends as they ensure that packing and food containers, for instance, are able to support relatively high stresses and keep adequate elongations at low temperatures.

### 3.4. Mechanical Properties of PBAT/PHB/Babassu Composites

**Figure 9:**
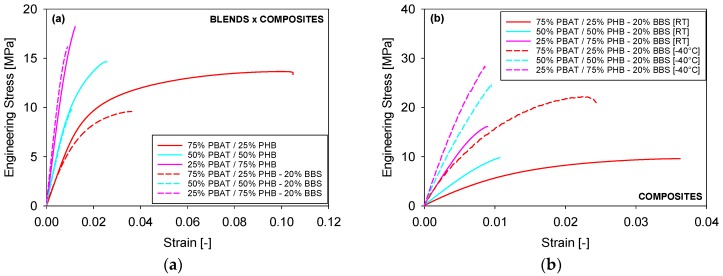
Representative stress-strain curves of PBAT/PHB/Babassu composites: (**a**) effect of babassu; (**b**) effect of temperature.

The effect of Babassu incorporation on the mechanical properties of PBAT/PHB blends tested at room temperature is displayed in Figure 9a. Babassu composites presented lower mechanical properties than their respective PBAT/PHB blends, i.e., reduction of 4.8 to 32.3% in tensile strength and from 26.0 to 66.3% in elongation at break. 

**Figure 10:**
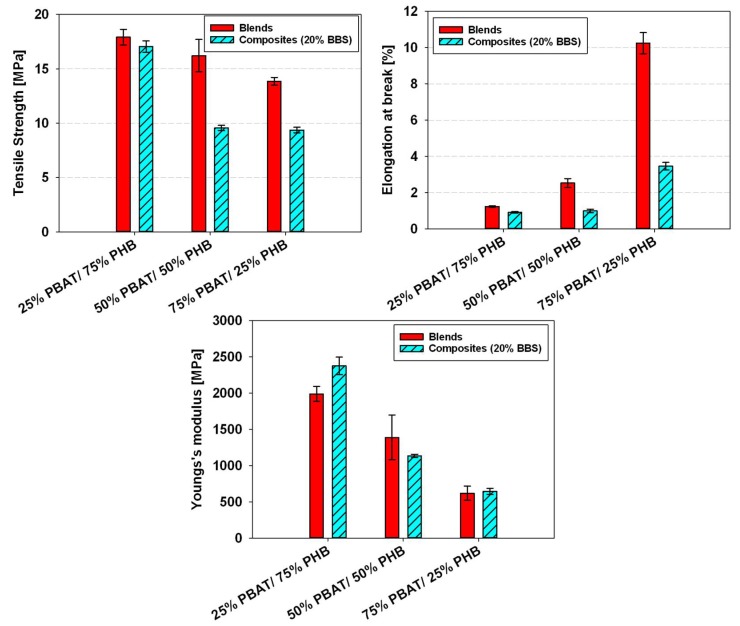
Comparison of mechanical properties of PBAT/PBHB blends and PBAT/PHB/Babassu composites tested at room temperature.

However, an increase in Young’s Modulus by 19.4% and 4.1% were observed for the 25%PBAT/75%PHB/20%BBS and 75%PBAT/25%PHB/20%BBS, respectively. These trends are presented in Figure 10. 

At lower temperature (−40 °C), both blends (Figure 7) and composites (Figure 9b) presented higher stiffness (i.e., Young’s modulus) and lower elongation at break. In this regard, Sorrentino et al. [29] investigated the effect of temperature on the mechanical properties of thermoplastic composites and similar trends to these reported in this work were observed. The authors also highlighted the importance of adhesion mechanisms between fillers and polymers on promoting better mechanical performances.

**Figure 11:**
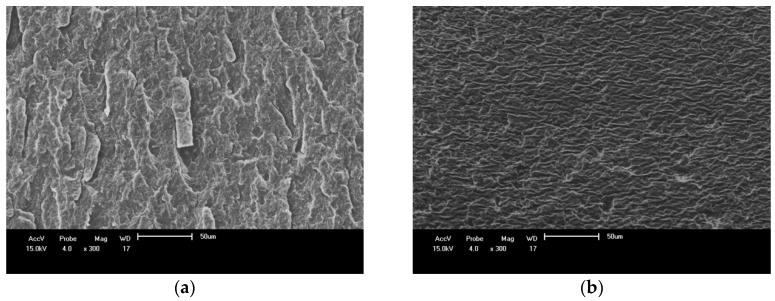
SEM images of cryo-fractured surface: (**a**) 25%PBAT/75%PHB; (**b**) 75%PBAT/25%PHB.

SEM images (Figure 11) show the presence of holes that might be responsible for the diminished tensile strength observed for the composites when compared to the neat blends. 

An aspect of fundamental importance for the performance of vegetal fiber–reinforced polymers are the mechanisms of water absorption, Chilali et al. [30] and Chen et al. [31] investigated this topic using aged composites. It appears that water absorption substantially affects the composites stiffness compared to unaged composites with a drop of about 45% in the tensile modulus after only two days of ageing. The water ageing causes a noticeable degradation of the tensile properties mainly due to the damage induced by fiber degradation and the weakening of the fiber matrix interface. 

From previous works it is known that Babassu filler is hydrophilic and absorbs humidity from the environment [32]. These mechanisms might interfere with the mechanical properties reported in the present contribution, i.e., Babassu filler might have absorbed water before/during processing and it would be contributing to the low performance of their mechanical behavior. Therefore, the present authors suggest a longer drying procedure and a composite processing directly after it. 

### 3.5. Scanning Electron Microscopy (SEM) of PBAT/PHB Blends

SEM images of 25%PBAT/75%PHB (left) and 75%PBAT/25%PHB blends (right) are shown in Figure 11. The SEM image of the blend with lower PBAT content (Figure 11a) is typical of brittle fracture with a seemly coarse surface and fracture propagation in a preferential direction. On the other hand, the SEM image for the 75%PBAT/25%PHB blend (Figure 11b) present regions with a more refined structure and no preferential direction. 

In this regard, it is likely that PBAT acts as a plasticizer for PHB. This assumption is supported by DSC data, in which blends with higher PBAT content have lower crystallinity. Crystalline phase is denser and more brittle than the amorphous one where deformation mechanisms easily take place. No phase coalescence is observed on these SEM images. 

Similar to the present work, Abdelwahab et al. [33] investigated the morphology of PLA/PHB blends plasticized with Lapol. The authors observed a coarse structure on the fracture surface with fractures propagating mainly along the same direction for the neat PLA/PHB blends indicating that the resistance to the development of fractures was quite low. However, upon addition of Lapol, the fracture surface of specimens became more refined with fractures propagating in different directions.

**Figure 12:**
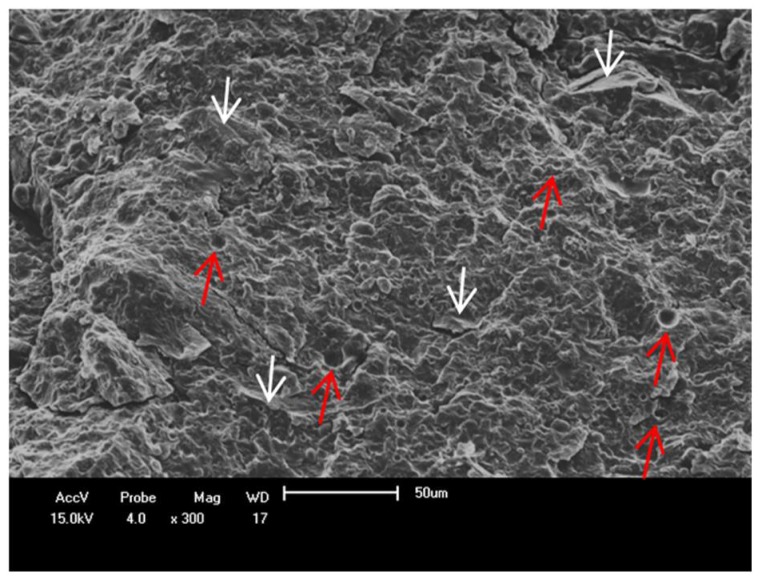
SEM images of cryo-fractured surface: 50%PBAT/50%PHB/20%BBS composites.

An SEM image of the fractured surface of 50%PBAT/50%PHB/20%BBS composite is shown in Figure 12, in which smooth oval hollow surfaces are observed (red arrows). These hollow surface are probably originated from bubbles due to improperly/inefficient drying or by chemical difference between polymers and fibers in composites as reported by Pérez-Fonseca et al. [34], Torres-Tello et al. [35], and Kurusu et al. [36]. In these works authors suggested that the chemical difference between polymers and fibers in composites could cause low compatibility between both materials generating voids or gaps at the interface, which lead to reduction in some mechanical properties, especially tensile strength. This agrees with the data observed in Figure 8 and Figure 10. Aggregates of Babassu particles, as well as poor filler/matrix adhesion are observed in Figure 12 (white arrows), which are also a source of lower mechanical performance. 

**Figure 13:**
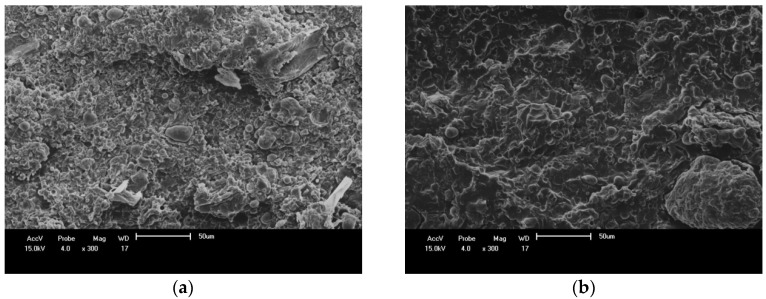
SEM images of testing-fractured surface of 50%PBAT/50%PHB/20%Babassu: (**a**) room temperature; (**b**) −40 °C.

Figure 13 shows SEM images for 50%PBAT/50%PHB/20%BBS composites fractured at room temperature (left) and at −40 °C (right). In both images no phase coalescence is observed. These images were acquired from the fractured surface specimens tested in Figure 9. The main difference in these samples is associated to the deformation mechanisms as specimens tested at room temperature deform more than those tested at −40 °C. 

The SEM image of 50%PBAT/50%PHB/20%Babassu composite tested at −40 °C (Figure 13b) seems to be coarser than the one tested at room temperature (Figure 13a). The presence of a more refined structure at room temperature might be an indication of fracture processes with more energy dissipation mechanisms leading to higher plastic deformation and elongation at break, which is validated by their tensile mechanical behavior. 

## 4. Conclusions

In the present work, PBAT/PHB/Babassu composites with 25, 50, and 75% of each polymer and 20% of Babassu were produced by melting extrusion. Blending of PBAT with PHB inhibited the crystallization of both polymers, whereas adding Babassu to PBAT/PHB blends did not significantly affect their melting behaviour. Incorporation of Babassu reduced tensile strength of its respective blends between 4.8 and 32.3%, and elongation at break between 26.0 and 66.3%. 

PBAT as highly ductile and low crystalline polymer may be seen as a crystallization tool control for PHB as well as a plasticizer to PBAT/PHB blends and PBAT/PHB/Babassu composites. As PBAT content increases: (i) elongation at break increases, and (ii) surface fracture becomes more refined indicating the presence of more energy dissipation mechanisms.

Babassu is a low-cost filler and PBAT/PHB/Babassu composites are biodegradable, environmental friendly, and cost effective. Products based on these compounds have great potential since their mechanical properties such as ductility, stiffness, and tensile strength are still suitable for several applications even at lower temperatures (−40 °C).

## Figures and Tables

**Table 1:** Compositions and codes of PBAT/PHB/Babassu composites.

Code	Mass (g)			
	PBAT	PHB	Babassu	Total
25%PBAT/75%PHB	68.75	206.25	0	275
50%PBAT/50%PHB	137.5	137.5	0	275
75%PBAT/25%PHB	206.25	68.75	0	275
25%PBAT/75%PHB/20%BBS	500	1500	500	2500
50%PBAT/50%PHB/20%BBS	110	1000	500	2500
75%PBAT/25%PHB/20%BBS	1500	500	500	2500

Note: BBS = Babassu.

**Table 2:** Selected samples, testing temperature, and fracture type of Scanning Electron Microscopy (SEM) analysis.

Sample	Testing Temperature ^1^	Fracture Type
25%PBAT/75%PHB	LN	cryogenic
75%PBAT/25%PHB	LN	cryogenic
50%PBAT/50%PHB/20%BBS	LN	cryogenic
50%PBAT/50%PHB/20%BBS	RT	tensile
50%PBAT/50%PHB/20%BBS	−40 °C	tensile

^1^ Liquid Nitrogen (LN) = −196 °C; Room Temperature (RT) = ~23 °C.

**Table 3:** Melting parameters of PBAT and PBAT/PHB blends—first peak.

PBAT/PHB	T_0.1%_	T_50%_	T_99.9%_	T_mp_	c_max_	τ_½_	∆H_m_	∆X_m_
(%)	(°C)	(°C)	(°C)	(°C)	(min^−1^)	(min)	(J/g)	(%)
100/0	89.35	117.68	142.61	93.64	0.335	2.83	18.64	16.35
75/25	87.68	115.75	136.30	86.65	0.351	2.81	4.43	3.89
50/50	101.92	119.13	133.04	101,37	0.522	1.72	1.76	1.54
25/75	-	-	-	-	-	-	-	-

Note: T_0.1%_ (°C) is the temperature to achieve 0.1% molten fraction, T_50%_ (°C) is the temperature to achieve 50% molten fraction, T_99.9%_ (°C) is the temperature to achieve 99.9% molten fraction, T_mp_ (°C) is the melting peak temperature, c_max_ (min^−1^) is the maximum melting rate, τ_½_ (min) is the melting half time (time to reach 50% molten fraction from the start of the event), ∆H_m_: (J/g) is the latent heat of melting, and ∆X_m_ (%) is the change in crystallinity during the event. The sign (-) indicate the absence of the peaks for that specific blend.

**Table 4:** Melting parameters of PHB and PBAT/PHB blends—second peak.

PBAT/PHB	T_0.1%_	T_50%_	T_99.9%_	T_mp_		c_max_	τ_½_	∆H_m_	∆X_m_
(%)	(°C)	(°C)	(°C)	(°C)		(min^−1^)	(min)	(J/g)	(%)
100/0	146.93	166.13	173.13	160.72	162.76	1.06	1.92	31.03	21.25
75/25	142.20	165.84	178.43	166.51	176.16	0.737	2.37	28.89	19.79
50/50	141.34	170.20	176.12	171.52	176.82	1.27	2.90	33.73	23.10
25/75	146.68	169.24	177.90	171.79	-	1.018	2.28	88.90	60.89

**Table 5:** Melting parameters of the second peak of PBAT/PHB/Babassu composites.

PBAT/PHB/BBS	T_0.1%_	T_50%_	T_99.9%_	T_mp_	c_max_	τ_½_	∆H_m_	∆X_m_
(%)	(°C)	(°C)	(°C)	(°C)	(min^−1^)	(min)	(J/g)	(%)
75/25/20	129.64	143.47	152.99	*148.81*	0.768	*1.39*	5.73	3.93
50/50/20	133.92	146.33	167.32	147.55	0.818	1.25	6.36	4.36
25/75/20	139.22	151.60	168.34	151.21	0.68	1.24	5.35	3.67
0/100/20	146.68	169.24	177.90	171.79	1.018	2.28	88.90	60.89
